# Polysaccharides: The Potential Prebiotics for Metabolic Associated Fatty Liver Disease (MAFLD)

**DOI:** 10.3390/nu15173722

**Published:** 2023-08-25

**Authors:** Qin Guo, Yun Li, Xin Dai, Bangmao Wang, Jie Zhang, Hailong Cao

**Affiliations:** 1 Tianjin Key Laboratory of Digestive Diseases, Department of Gastroenterology and Hepatology, Tianjin Institute of Digestive Diseases, National Key Clinical Specialty, General Hospital, Tianjin Medical University, Tianjin 300052, China; guoqin11@tmu.edu.cn (Q.G.); liyun6706@tmu.edu.cn (Y.L.); daixin1986@tmu.edu.cn (X.D.); mwang02@tmu.edu.cn (B.W.); 2 Department of Pharmacy, General Hospital, Tianjin Medical University, Tianjin 300052, China

**Keywords:** polysaccharides, prebiotic, metabolic associated fatty liver disease

## Abstract

Metabolic (dysfunction) associated fatty liver disease (MAFLD) is recognized as the most prevalent chronic liver disease globally. However, its pathogenesis remains incompletely understood. Recent advancements in the gut-liver axis offer novel insights into the development of MAFLD. Polysaccharides, primarily derived from fungal and algal sources, abundantly exist in the human diet and exert beneficial effects on glycometabolism, lipid metabolism, inflammation, immune modulation, oxidative stress, and the release of MAFLD. Numerous studies have demonstrated that these bioactivities of polysaccharides are associated with their prebiotic properties, including the ability to modulate the gut microbiome profile, maintain gut barrier integrity, regulate metabolites produced by gut microbiota such as lipopolysaccharide (LPS), short-chain fatty acids (SCFAs), and bile acids (BAs), and contribute to intestinal homeostasis. This narrative review aims to present a comprehensive summary of the current understanding of the protective effects of polysaccharides on MAFLD through their interactions with the gut microbiota and its metabolites. Specifically, we highlight the potential molecular mechanisms underlying the prebiotic effects of polysaccharides, which may give new avenues for the prevention and treatment of MAFLD.

## 1. Introduction

Non-alcoholic fatty liver disease (NAFLD) has emerged as a prevailing form of chronic liver disease on a global scale, making it a significant health concern. With a staggering estimated prevalence exceeding 25% globally, a closer examination revealed the Middle East and South America as the regions with the highest rates, standing at an impressive 32% and 31%, respectively. In stark contrast, Africa portrayed the lowest rates, registering a mere 14% [1,2]. NAFLD encompasses a spectrum of pathological conditions, ranging from simple hepatic steatosis to more severe forms such as Non-alcoholic Steatohepatitis (NASH), cirrhosis, and hepatocellular carcinoma (HCC) [3]. The progression to cirrhosis is a robust predictor of liver-related mortality [4]. In a groundbreaking development of 2020, a consensus reached by a distinguished international panel of experts brought forth a proposal to rename the disease formerly known as NAFLD to the more descriptive term-Metabolic (dysfunction) associated fatty liver disease (MAFLD) [5]. The diagnosis of MAFLD mandates the identification of hepatic steatosis through meticulous histopathological examination. Additionally, at least any one of the following three criteria must be met: the individual exhibits overweight/obesity has type 2 diabetes mellitus (T2DM), or presents evidence of metabolic dysregulation. This novel set of criteria places paramount importance on the presence of metabolic dysfunction, while the exclusion of patients with alcohol consumption or other chronic liver diseases is no longer necessary for diagnosis [6]. A study showed that the new definition of MAFLD could distinguish more patients with clinical risk, as metabolic dysfunction significantly heightened the susceptibility to cirrhosis and HCC [7,8]. In this article, we adopt the term MAFLD to represent NAFLD, a decision driven by expertise and adherence to academic rigor.

The pathogenesis of MAFLD is a complex and multifactorial process. In 1998, the “two-hit” hypothesis was proposed to describe the theory. According to this hypothesis, the first hit involves an increase in intrahepatic fat accumulation, linked to factors such as insulin resistance, a sedentary lifestyle, and high-calorie intake. The second hit occurs when lipid-induced oxidative stress, cytokine-mediated inflammation, mitochondrial dysfunction, apoptosis, necroinflammation, and fibrosis come into play, ultimately resulting in the progression to cirrhosis and HCC. However, the two-hit hypothesis alone is insufficient to fully elucidate the complexity of MAFLD progression in humans. A more comprehensive understanding can be achieved through the “multi-hit” hypothesis, which considers the intricate interplay between genetic and environmental factors, as well as the crosstalk between different organs. This hypothesis provides a more reasonable framework for comprehending the development of MAFLD in humans in the present day [9].

Polysaccharides, as a prominent component of the human diet, have garnered significant attention due to their potential impact on metabolic processes. While other dietary components also play a role, the focus of this study is on polysaccharides due to their abundant presence and their close interaction with the gut microbiota. Polysaccharides are polymeric carbohydrate macromolecules composed of over 10 monosaccharide molecules joined together by glycosidic bonds. These remarkable molecules are abundantly found in fruits, vegetables, fungi, Chinese herbal medicine, and algae. Carbohydrates could generally be classified into two groups. The first group comprises storage carbohydrates with starch being the representative substance. Starch is primarily digested and absorbed in the small intestine to provide a valuable source of calories for the human body. The other consists of cell wall polysaccharides and also known as non-starch polysaccharides (NSPs). Unlike storage carbohydrates, NSPs cannot be directly digested by the human body and are instead major contributors to fermentable dietary fiber. In this article, our focus centers on NSPs. Previous studies have underscored the diverse biological activities that polysaccharides possess, including anti-inflammatory [10], anti-tumor [11], immune-regulating and antioxidant properties [12,13]. Supplementation of polysaccharides held great potential as an effective treatment for metabolic-related diseases, such as MAFLD, T2DM, and atherosclerosis (AS) [14,15]. Especially, MAFLD-related features (weight, blood glucose, blood lipids, steatosis, hepatocyte inflammation) can be improved. The accumulating evidence strongly supports the inhibitory effects of polysaccharides on the development and progression of MAFLD [16]. The entire polysaccharide molecule demonstrated significant potential in influencing the course of MAFLD, as supported by findings from the literature. However, it is the specific oligosaccharide components embedded within the polysaccharide structure that played a central role in mediating these effects. The structural characteristics of these oligosaccharides, such as chain length, glycosidic linkages, and sugar residues, were crucial in determining their bioactivity. Notably, certain oligosaccharide structures derived from Poria cocos, Bletilla striata, and Radix Puerariae thomsonii polysaccharides exhibited remarkable affinity towards receptors, enzymes, and signaling pathways that are intricately involved in the progression of MAFLD. Through these interactions, these specific oligosaccharides unleashed therapeutic effects that hold promise in alleviating the impact of MAFLD [17,18,19]. Furthermore, recent research has shed light on the capacity of polysaccharides to modulate gut microbiota composition and reduce blood lipid levels. Dietary fiber, a derivative of polysaccharides, has also been shown to alleviate liver steatosis through its regulation of the gut microbiota [20]. Therefore, this narrative review targets to explore polysaccharides and their interplay with the gut microbiota, which holds tremendous potential for innovative therapeutic approaches in managing MAFLD and associated metabolic syndromes.

## 2. Polysaccharides against MAFLD

### 2.1. MAFLD and Intestinal Dysbiosis

In the last five years, researchers worldwide have been dedicating significant attention to the field of intestinal dysbiosis and its role in the development of MAFLD, recognizing the interconnectedness of the gut and liver in both anatomy and function [21]. The concept of the gut-liver axis, a distinctive relationship, was introduced by Marshall in 1998. Recent studies have indicated that MAFLD can be influenced by intestinal dysbiosis, with several investigations confirming alterations in the abundance of gut microbial communities in MAFLD patients. Unfortunately, due to variations in sample sizes, geographical locations, and individual characteristics of gut microbiota, the results were inconsistent and contradictory [22]. Nevertheless, emerging evidence strongly suggests a convincing correlation between gut microbiota and metabolic dysfunction-related diseases. Multiple studies utilizing 16S rRNA sequencing have consistently reported a reduction in diversity at the genus level, with specific flora modifications associated with different clinical stages of MAFLD, contributing to distinct microbiome features [23,24]. Overall, the most consistent result from patients with MAFLD was that the increased abundance of bacteria with pro-inflammatory effects, such as *Proteobacteria*, *Fusobacteria,* and *Verrucomicrobia*, alongside a decrease in the density of *Bacteroidetes*, *Firmicutes*, *Ruminococcaceae*, *Anaerospacter*, *Coprococcus,* and *Eubacterium* [25,26,27]. During the steatohepatitis stage, contradictory studies showed that either the increases in gut *Bacteroides* abundance or the decreases in *Prevotella* owning anti-inflammatory properties promoted liver inflammation of adult MAFLD [28]. These variations underscore the intricate nature of the gut-liver axis and emphasize the necessity for additional research to elucidate the precise mechanisms underlying the interplay between gut microbiota and liver inflammation. The fecal microbiome exhibits distinct features as the disease progressed from steatosis and inflammation to fibrosis. Notably, *Ruminococcus* was independently associated with hepatic fibrosis. According to other studies, metagenomic sequencing showed that significant fibrosis was closely related to the increased abundance of *Bacteroides vulgatus* and *Escherichia coli* [29]. In addition, the genus *Escherichia_Shigella* and the *Enterobacteriaceae* family tended to be more abundant in patients with advanced fibrosis [27].

An intriguing finding is the potential alleviation of hepatic steatosis through the use of antibiotics, as demonstrated in both mouse models and clinical trials involving high-fat diets [30,31]. Additionally, fecal microbiota transplantation (FMT) has shown promise in ameliorating the progression of MAFLD, with FMT from healthy donors restoring gut barrier integrity, mitigating histological liver changes, and reducing endotoxemia in patients [32,33]. An abundance of data has presented convincing evidence that strongly substantiates the crucial involvement of the gut microbiome in the pathogenesis of MAFLD. Besides various pathogenic elements related to influence on gut microbiota, the collection and processing course of fecal samples were proven to emerge variances and inaccuracies in the taxa of the intestinal microbiota [34]. For these reasons, a larger sample size, multinationals and multiracial clinical research, and more accurate experimental methods seem to be crucial ways to investigate the interrelation and eliminate contradictory findings between gut microbiota and MAFLD.

The potential mechanisms include small intestinal bacterial overgrowth (SIBO), disruption of intestinal barriers, bacterial translocation, elevated serum lipopolysaccharide (LPS) levels, and subsequent inflammatory response [35]. When the injuries of intestinal mucosal barriers and increased intestinal permeability occur, transporting LPS produced by intestinal bacteria into the bloodstream promotes the progression of MAFLD [36]. Moreover, the other metabolites of microbiota include short-chain fatty acids (SCFAs), bile acids (BAs), trimethylamine oxide (TMAO), endogenous ethanol (EnEth), and ammonia exerting their influence on MAFLD through diverse mechanisms. SCFAs, a vital source of energy for the host, take a key role in protecting the integrity of intestinal epithelial, regulating physiological function, and preventing obesity [37]. BAs actively participate in cholesterol metabolism within the hepatic system and improve anti-inflammatory ability, hepatic glucose, and lipid metabolism through microbiota-induced signaling pathways involving farnesoid X receptor (FXR) and Takeda G protein-coupled receptor 5 (TGR5) [38]. The disruption of SCFAs or BAs homeostasis can weaken their positive effects on liver health. Trimethylamine (TMA) is a sort of choline-derived product metabolized by gut microbiota and subsequently oxidized in the liver to produce TMAO. Increased levels of circulating TMAO have been linked to an elevated body mass index, impaired glucose tolerance, and hepatic triglyceride accumulation [39]. EnEth from bacteria producing ethanol including *Bacteroides fragilis*, *Escherichia* and *Enterobacteriaceae* enters the liver. EnEth not only activates the cytochrome P450 family 2 subfamily E polypeptide 1 (CYP2E1) enzyme, leading to oxidative damage and necrosis of hepatocytes but also induces lipid deposition. Additionally, EnEth causes injury to intestinal tight junctions (TJs) and triggers inflammation, thereby accelerating the progression of MAFLD [40]. Additionally, there is accumulating evidence that urea cycle disorder-induced hyperammonemia is related to suboptimal liver function which involves the progression of fibrosis in MAFLD [41]. On the whole, gut microbiota and metabolites may be opening up potential therapeutic opportunities and clues in the management of MAFLD.

### 2.2. Polysaccharides Regulate MAFLD via Gut Microbiota

Polysaccharides pose a challenge to digestion by gastrointestinal enzymes because of the multiple interconnecting ways among the monosaccharide units inducing complex chemical structures. As a result, most of the polysaccharides are fermented by bacterial enzymes produced by the gut microbiota in an anaerobic environment when reach the colon [42]. The fermentation process gives rise to metabolites such as SCFAs and succinate, which play a foundational part in regulating the composition and function of the gut microbiome [43]. The interaction between polysaccharides and the gut microbiota holds significant implications for maintaining a healthy intestinal microecology. For instance, β-glucan increased intestinal lactic acid content by increasing the abundance of *Lactobacillus* and *Bifidobacterium*, which promotes a favorable gut environment [44]. Plantago asiatica L polysaccharides were shown to promote the abundance of *Lactobacillus fermentum* and *Bacteroides ovatus*, while also increasing the production of acetic acid, butyric acid, and propionic acid in intestines [45]. In addition to these examples, other polysaccharides have demonstrated their ability to restore the flora disorder caused by a high-fat diet (HFD) and prevent or treat related diseases. Polysaccharides derived from Chenopodium quinoa, a traditional Inca food, attenuated hyperlipidemia by reducing the relative abundance of bacteria *Firmicutes/Bacteroides* ratio in HFD-fed mice, as well as the abundance of *Proteobacteria* and *Desulfovibrio* [46]. Modified apple polysaccharides (MAP) were effective in restoring the imbalance of gut microbiota caused by increasing the levels of *Bacteroides* [47]. Polysaccharides from Momordica charantia also exhibited an anti-obesity effect in HFD-fed mice by enhancing the abundance of *Actinobacteria* and decreasing detrimental *Proteobacteria* and *Helicobacter* pylori [48]. Similarly, Nigella sativa seed polysaccharides (NSSP) alleviated symptoms of T2DM in mice induced by an HFD and streptozotocin (STZ), notably by enhancing the presence of gut microbiota from the *Muribaculaceae* family and *Bacteroides* [49]. Flaxseed polysaccharides attenuated metabolic syndrome (MetS) in mice fed an HFD by promoting the growth of beneficial probiotics *Akkermansia* and *Bifidobacterium*, reducing the obesity-associated bacteria such as *Oscillospira* and *Odoribacteraceae* [50]. These researches well suggested that different molecular structures make polysaccharides have various effects on the composition of gut microbiota. Despite this, all of these polysaccharides demonstrated the ability to ameliorate the dysbiosis of intestinal flora induced by an HFD. This suggests that polysaccharides have the potential to act as prebiotics, offering a promising approach to mitigate MAFLD, a condition primarily triggered by HFD consumption (Table 1).

Food sources rich in polysaccharides or the extract of polysaccharides thereof have been observed to have a positive mitigation effect on the development of MAFLD, and the physiological process is believed to involve the dynamic interaction between the liver and the intestinal tract. In one study, *Hirsutella sinensis* polysaccharides (HSM) were extracts of anamorph from cordyceps (fungi polysaccharides) reducing the accumulation of lipid droplets and liver cell hypertrophy via modulating gut bacteria *Parabacteroides goldsteinii* in HFD-induced mice. HSM supplementation was shown to reduce serum triglyceride levels and downregulation of genes associated with lipogenesis, lipid transport, and uptake. Conversely, gene expression related to hepatic β-oxidation and thermogenesis were up-regulated in the HSM-treated group. Additionally, the similar anti-MAFLD effect observed with FMT was abolished by neomycin treatment, indicating the crucial role of gut microbiota in modulating MAFLD [51]. Polysaccharides from edible Grifola frondose(GFP, fungi polysaccharides) were shown to modulate the dysregulated microbiota associated with lipid metabolism disorders, and particularly enhance the proliferation of advantageous bacteria *Helicobacter*, *Intestinimonas,* and *Barnesiella* while decreasing the prevalence of *Clostridium*, *Butyricicoccus*, and *Turicibacter* [53]. Another study found that GFP could reduce the *Firmicutes*/*Bacteroidetes* ratio, with lower energy-harvesting capacity from polysaccharides, leading to a decreased generation of lipids and consequently alleviating hepatocyte steatosis and liver cell injury [52]. These studies illustrated the potential of GFP intervention in inhibiting the progression of MAFLD probably through modulating the gut microbiota. Walnut green husk polysaccharides (WGHP, plant polysaccharides) reversed colonic tissue injury, improve the expression of proteins involved in TJs., and increase the abundance of *Prevotellaceae* and *Allobaculum* in HFD-fed rats. The results suggested that WGHP could prevent liver damage, obesity, and inflammation by gut-liver axis [55]. The supplementation of polysaccharides was shown to regulate the disorder of gut microbiota and exert anti-MAFLD effects through various mechanisms.

## 3. Possible Mechanisms of Polysaccharides on MAFLD

**Figure 1:**
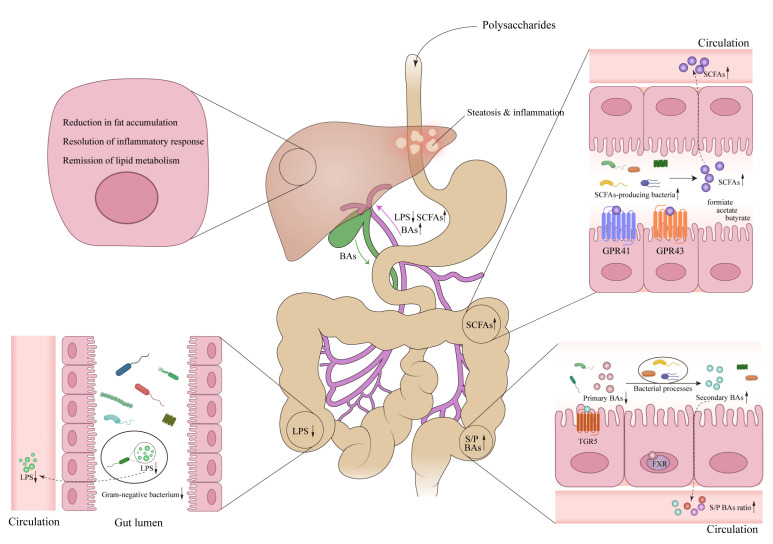
The preventive and therapeutic effects of polysaccharides in metabolic associated fatty liver disease (MAFLD) via enterohepatic axis. Polysaccharides, upon entering the intestinal tract, regulate the gut microbiota, thereby influencing the metabolism of LPS, SCFAs, and BAs. This ultimately leads to changes in the levels of these metabolites entering the liver through the portal vein, resulting in improved hepatic steatosis and inflammation levels in liver cells. Abbreviations: LPS, Lipopolysaccharide; SCFAs, Short chain fatty acids; GPR41, G-protein-coupled receptor 41; GPR43, G-protein-coupled receptor 43; BAs, bile acids; S/P BAs, secondary/primary bile acids; FXR, farnesoid X receptor; TGR5, Takeda G protein-coupled receptor 5. ↓, down-regulation after administration of polysaccharides; ↑, up-regulation after administration of polysaccharides.

The potential of polysaccharides to improve MAFLD by interacting with the gut microbiota and its metabolites offers a promising avenue for therapeutic intervention. Our findings highlight the ability of polysaccharides to shape the gut microbiota composition, favoring the proliferation of beneficial bacteria while diminishing the abundance of detrimental species. This reshaping of the microbial landscape results in a decrease in the synthesis and movement of endotoxins, which are pivotal contributors to the inflammatory processes and liver damage observed in MAFLD. Moreover, polysaccharides promote the production of SCFAs, which have shown anti-inflammatory and metabolic benefits within the liver. Polysaccharides could also influence bile acid metabolism, an essential role in regulating lipid metabolism and the development of hepatic steatosis. Collectively, these findings underscore the elaborate mechanism of polysaccharides offering a novel approach to target the gut microbiota and its metabolites for the management of MAFLD (Figure 1).

### 3.1. Protecting Intestinal Barriers and Reducing Endotoxemia

**Figure 2:**
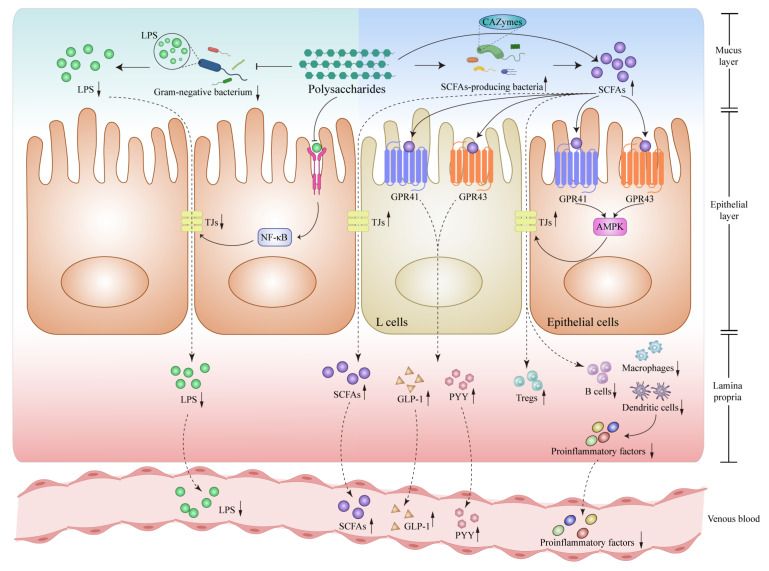
Polysaccharides modulate Lipopolysaccharide (LPS) and short-chain fatty acids (SCFAs) related signaling pathways in intestines. Polysaccharide intervention attenuates the activation of the TLR4-NF-κB-tight junction protein signaling pathway located on the surface of epithelial cells, reducing the presence of LPS-producing bacteria within the intestines, decreasing LPS levels and reducing the entry of LPS into the bloodstream by reinforcing TJs. Polysaccharide intervention increases the production of SCFAs to activate GPR41 and GPR43 receptors, which further stimulate the release of GLP-1 and PYY by intestinal L cells, meanwhile, it could also bolster TJs and fortify the integrity of the intestinal barrier through AMPK-related signaling pathways, enhancing the population of Tregs while reducing B cells, macrophages and dendritic cells to reduce proinflammatory factors into the bloodstream. All of the above possible mechanisms are summarized from the current references. Abbreviations: LPS, Lipopolysaccharide; TLR4, Toll-like receptor 4; NF-κB, nuclear factor kappa-B; TJs, tight junctions; SCFAs, Short chain fatty acids; CAZymes, carbohydrate-activated enzymes; GPR41, G-protein-coupled receptor 41; GPR43, G-protein-coupled receptor 43; AMPK, AMP-activated protein kinase; GLP-1, glucagon-like peptide 1; PYY, peptide YY; Tregs, regulatory T cells. ↓, down-regulation after administration of polysaccharides; ↑, up-regulation after administration of polysaccharides.

As we mentioned before, SIBO, especially the gram-negative bacteria produce LPS which is one of the pathogen-associated molecular pattern (PAMP) molecules. As intestinal permeability increases, bacterial translocation occurs, allowing LPS to penetrate the portal vein. Subsequently, LPS triggers the activation of pattern recognition receptors, including toll-like receptors (TLRs), and inflammasome in the liver, inducing hepatic immunologic and inflammatory responses. One classical signaling pathway is the TLR4-mediated signaling pathway which recognizes bacterial LPS signals, subsequently activating nuclear factor kappa-B gene binding (NF-κB) and promoting the production of proinflammatory cytokines, which significantly contribute to the progression of MAFLD [66] (Figure 2).

Lycium barbarum polysaccharide (LBP, plant polysaccharides) fed rats showed a protective function in MAFLD. This was evidenced by an observed increase in the abundance of *Deferribacteracea* and a decrease in the presence of *Enterococcaceae* and *Proteobacteria*. Moreover, LBP supplementation led to the upregulation of ZO-1 and occludin protein expressions, maintaining the regular structural morphology of TJs. Importantly, LBP significantly reduced plasma levels of LPS and D-Lactate in plasma compared to the HFD-fed group. Investigating the underlying mechanism, LBP safeguarded intestinal barrier integrity, hampered the growth of LPS-producing bacteria, gram-negative aerobic bacteria, and reduced the systemic release of LPS, thereby mitigating hepatic inflammation [56]. Lentinan, a plant polysaccharide extracted from *shiitake mushrooms* (fungi polysaccharides), significantly improved intestinal microbiota disorder in HFD mice, with an augmented abundance of *Actinobacteria* and a reduction in *Proteobacteria* and *Epsilonbacteraeota* at the phylum level. Lentinan also strengthened the intestinal barriers and decreases serum LPS levels, specifically down-regulates the expression of hepatic LPS-binding protein (LBP), TLR4, and downstream proinflammatory cytokines and inhibited the decline of antioxidants such as nuclear factor erythroid 2-related factor 2 (Nrf2), heme oxygenase-1 (HO-1), NADPH: quinone oxidoreductase 1 (NQO1) and glutamate cysteine ligase catalytic subunit (Gclc). These effects attenuated the NFκB-PTP1B-Akt-GSK3β signaling pathway in the liver, thereby offering potential therapeutic benefits of MAFLD [58]. The administration of *Salvia miltiorrhiza* polysaccharide (plant polysaccharides) to mice noticeably increased the ratio of *Bacteroidetes/Firmicutes* by 34.04%. This plant polysaccharide increased acetate and butyrate with the decrease in serum LPS to alleviate hepatic inflammation compared to the HFD group. These findings proposed a co-regulation mechanism involving multiple pathways for the strategies to combat MAFLD [59].

### 3.2. Improvement of the Levels of SCFAs

Microbial metabolites have emerged as significant contributors to the progression of MAFLD. Non-digestible dietary fibers undergo fermentation by gut bacteria to produce short-chain fatty acids, including acetate, propionate, and butyrate. SCFAs not only provide extra energy to the host but also hold pivotal roles in regulating lipogenesis and gluconeogenesis. By influencing these metabolic pathways, SCFAs exert profound effects on managing MAFLD [67].

The intriguing relationship between specific gut bacteria and carbohydrate digestion has garnered considerable attention recently. These bacteria produce carbohydrate-activated enzymes (CAZymes) which could affect polysaccharides degradation and modification. CAZymes could be categorized into two types based on their enzymatic reaction mechanism: glycoside hydrolases (GHs) and polysaccharide lyases (PLs). Through their enzymatic activities, these CAZymes facilitate the intricate metabolism of complex polysaccharides by bacteria, resulting in the production of metabolically significant molecules such as SCFAs, LPS, and carbon monoxide. These metabolites, along with the synthesis of cell components, contribute to a multitude of metabolic processes within the body [68]. Polysaccharides and gut microbiome interact with each other, polysaccharides serve as essential substrates for the production of SCFAs, playing a vital role in the metabolic process. A variety of studies highlighted the role of SCFAs in activating two G-protein-coupled receptors (GPRs) GPR41 and GPR43 protect MAFLD, showcasing their impact on various tissues including the intestines, liver, and white adipose tissue (WAT). As reported, activation of GPR41 by propionate and butyrate led to enhanced peptide YY (PYY) secretion, stimulating satiety and decreasing mobility of gastrointestinal in enteroendocrine cells [69]. Otherwise, SCFAs particularly acetate and propionate increased concentrations of the hormone glucagon-like peptide 1(GLP-1) in the blood plasma through GPR43 to control blood glucose and suppress appetite [70]. Both two receptors appear to have beneficial effects on weight control, while some studies stated that SCFAs may increase energy harvest and lead to weight gain [71]. In the context of MAFLD, butyrate entering the hepatocytes via the portal vein modulated disease progression by activating AMP-activated protein kinase (AMPK), decreasing the activity and expression of proliferator-activator receptor-γ (PPAR-γ) and Sterol regulatory element-binding protein 1C (SREBP-1c) in the liver. Consequently, hepatic lipogenesis was impeded, insulin resistance was reduced, and oxidative stress and inflammation were alleviated [72,73]. Moreover, emerging research identified the underlying ability to inhibit MAFLD development at the epigenetic level. In adipocytes, SCFAs prevented fat accumulation and increased thermogenesis in WAT. These positive changes were related to the activation of PPAR-γ and browning of WAT [74]. While the vital signal molecule looks like playing a controversial role in patients with MAFLD, the above evidence illustrates that SCFAs exert potential beneficial effects on body metabolism and hepatic steatosis (Figure 2).

In a word, the presence of polysaccharides and the gut microbiome synergistically contribute to the production of SCFAs by bacteria, playing a pivotal role in regulating metabolic processes and their impact on MAFLD. MDG-1 polysaccharide, an inulin-type β-D-fructan extract derived from Ophiopogon japonicus roots (plant polysaccharides), exhibited significant inhibitory effects on lipid accumulation, hepatic injury, and macrovesicular steatosis in HFD-fed mice. Through its mechanisms of action, MDG-1 showed a positive influence on MAFLD by rebalancing the composition of gut bacteria affected by the HFD and boosting the levels of SCFA-producing microbial genera including *Butyricimonas* and *Roseburia*. Compared to the HFD group, the group treated with MDG-1 exhibited a notable increase in total SCFAs especially acetic acid and valeric acid, and the lipid metabolism genes AMPK, AMPK phosphorylation, and SCFAs receptors GPR41 and GPR43 were up-regulated. Generally speaking, MDG-1 increased SCFAs through intestinal bacteria, subsequently activated the AMPK-related hepatic lipid uptake signaling pathway, and restored normal lipid metabolism to offer a promising therapeutic strategy for MAFLD [61]. Another polysaccharide, Astragalus polysaccharides (APS) was extracted from the traditional Chinese herb Astragalus mongholicus Bunge (plant polysaccharides). APS inhibited the expressions of lipid metabolism gene hepatic glucokinase (GCK), hepatic fatty acid synthase (FASN), and cluster of differentiation 36 (CD36) protein, meanwhile up-regulating the expression of recombinant carnitine palmitoyltransferase 1α (Cpt1α) and peroxisome proliferator-activated receptor α (PPARα) mRNA. Metagenomics revealed that APS enriched *Desulfovibrio vulgaris* from *Desulfovibrio* genus effectively releasing HFD-induced hepatic steatosis in mice. This beneficial effect was attributed to the increase in acetic acid levels, rather than propionic acid or butyric acid, in both serum and feces [62].

### 3.3. Change the Composition of Bile Acids

**Figure 3:**
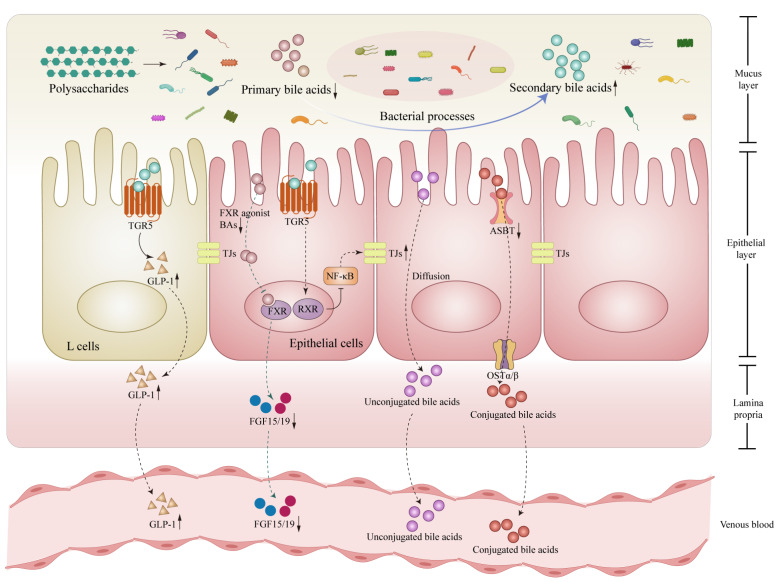
Polysaccharides modulate bile acids (BAs) metabolism through microbiota in the intestines. Polysaccharides promote the conversion of primary BAs to secondary BAs by the structural transformation effect of microbiota, which leads to a decrease in the binding of primary BAs to the FXR, following a lessened production of FGF15/19 in the gut. Correspondingly, the activation of the TGR5 by secondary BAs is enhanced, stimulating the production of GLP-1 by intestinal L cells, strengthening the activation of RXR in intestinal epithelial cells, thereby blocking the NF-κB-TJs inflammatory-related signaling pathway and enhancing intestinal barrier function, This also results in a downregulation of specific transporter such as ASBT, reducing the influx of conjugated bile acids into the bloodstream and promoting their excretion from the gut. Abbreviations: BAs, bile acids; FXR, farnesoid X receptor; RXR, retinoid X receptor; TGR5, Takeda G protein-coupled receptor 5; NF-κB, nuclear factor kappa-B; FGF15/19, fibroblast growth factor 15/19; GLP-1, glucagon-like peptide 1; ASBT, apical sodium-dependent bile acids transporter; OSTα/β, organic solute transporter alpha-beta; TJs, tight junctions. ↓, down-regulation after administration of polysaccharides; ↑, up-regulation after administration of polysaccharides.

**Figure 4:**
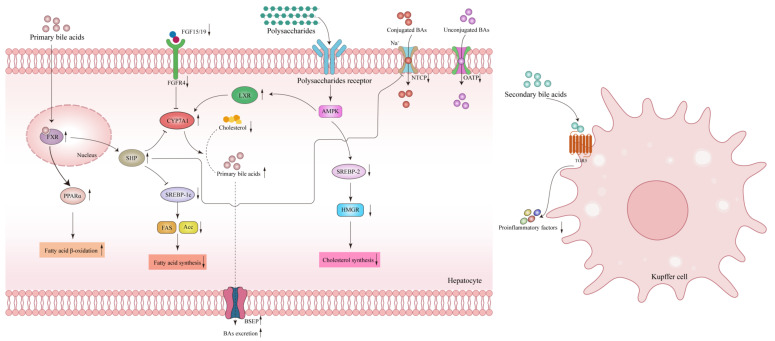
Polysaccharides modulate liver bile acids (BAs)-related signaling pathways of metabolic associated fatty liver disease (MAFLD). Polysaccharides impact the bile acid regulation in the liver: The secondary bile acids stimulate the surface TGR5 receptor on Kupffer cells, reducing pro-inflammatory factors and alleviating liver inflammation. Primary bile acids trigger the nuclear receptor FXR in hepatocytes to enhance the β-oxidation of fatty acids by up-regulating the expression of PPARα, inhibiting the expression of SREBP-1c through the SHP, thereby reducing fatty acid synthesis. By attenuating the binding between weakened FGF15/19 molecules from the intestines and the FGFR4/β-Klotho receptor complex, polysaccharides alleviate the inhibition of CYP7A1 and promote cholesterol conversion to bile acids. In addition, polysaccharides on the one hand bind to its’ autoreceptor, increasing CYP7A1 expression through the activation of the AMPK-LXR signaling pathway, on the other hand, down-regulate cholesterol synthesis through the AMPK-SREBP-2-HMGR related signaling pathway, reducing BAs absorption related transporters NTCP and OATP while increasing excretion related transporter BSEP. Abbreviations: BAs, bile acids; FXR, farnesoid X receptor; LXR, liver X receptor; TGR5, Takeda G protein-coupled receptor 5; FGF15/19, fibroblast growth factor 15/19; PPARα, peroxisome proliferator-activated receptor α; FGFR4, fibroblast growth factor receptor 4; CYP7A1, cholesterol 7α-hydroxylase; AMPK, AMP-activated protein kinase; SHP, recombinant small heterodimer partner; SREBP-1c, Sterol regulatory element-binding protein 1C; SREBP-2, Sterol regulatory element-binding protein-2; HMGR, 3-hydroxy-3-methylglutaryl-CoA reductase; NTCP, Na^+^-taurocholate co-transporting polypeptide; OATP, organic anion transport protein; BSEP, bile salt export pump. ↓, down-regulation after administration of polysaccharides; ↑, up-regulation after administration of polysaccharides.

As amphipathic hydroxylated steroid molecules, bile acids (BAs) are synthesized through the breakdown of cholesterol in the liver and subsequently released into the duodenum from the gallbladder. The production of primary BAs is catalysed by rate-limiting enzyme cholesterol 7α-hydroxylase (CYP7A1), oxysterol 7α-hydroxylase (CYP7B1), and sterol-27-hydroxylase (CYP27A1). The gut microbiota remains a key factor in the modification of bile salts, transforming primary into secondary BAs through bacterial reactions. The conjugated bile acids are uncoupled by bile salt hydrolase (BSH) produced by intestinal bacteria to become unconjugated BAs. Following, unconjugated BAs become secondary BAs such as deoxycholic acid (DCA), lithocholic acid (LCA), and ursodeoxycholic acid via undergoing additional transformations such as C-3, C-7, and C-12 positions epimerization along with 7α-dehydroxylation. In the enterohepatic circulation, the majority of BAs are reabsorbed into the portal vein for recycling. However, a small fraction of BAs was found to act as signaling molecules in the bloodstream [75,76,77]. Preclinical and clinical investigations have confirmed BAs interacting with BAs responsive receptors such as FXR, TGR5, and liver X receptor (LXR) could modulate the development of MAFLD [78]. On the currently available pieces of evidence from references, the possible related mechanisms that have been proposed to explain the involvement of BAs in the intestines (Figure 3) and liver (Figure 4) are as follows.

BAs generally play a significant role in countering MAFLD in the following three aspects: Firstly, in terms of lipid metabolism, primary BAs such as CDCA activate the FXR receptor and induce the release of hepatic fibroblast growth factor 21 (FGF21) to facilitate the breakdown of fatty acids and energy production in adipose tissue [79]. FXR activation also leads to a decrease in lipoprotein lipase within very low-density lipoprotein (VLDL), resulting in reduced plasma triglyceride levels [80]. In humans, an elevation in low-density lipoprotein cholesterol (LDL-C) may occur due to FXR-dependent inhibition of cholesterol-to-bile acid conversion, leading to increased liver cholesterol deposition [81]. FXR activation also reduces adipogenesis by inhibiting the expression of liver SREBP-1c via recombinant small heterodimer partner (SHP) and intestinal-derived FGF15/19. This process enhances the β-oxidation of fatty acids, decreases fatty acids synthesis, and affects cholesterol transport [82]. Secondly, BAs impact glucose metabolism. FXR signaling directly contributes to increased insulin sensitivity, promotes hepatic glycogen synthesis, and inhibits gluconeogenesis in the intestines, liver, and pancreas [83,84]. Secondary BAs DCA could bind to TGR5 receptors, triggering the TGR5-cAMP dependent pathway and increasing generation of the GLP-1 in intestinal endocrine L cells, thereby augmenting insulin secretion and regulating glucose metabolism homeostasis [85]. Thirdly, BAs release inflammation response. Activation of TGR5 provides valuable protection against LPS-NF-κB-induced inflammation. TGR5 activation inhibits the production of cytokines such as interleukin-6 (IL-6), IL-1A, IL-1B, tumor necrosis factor (TNF), and inducible nitric oxide synthase (iNOS) via the cAMP-PKA-dependent signaling pathway, effectively reducing inflammation in liver Kupffer cell [86,87]. Furthermore, TGR5 activation also hampers the activation of NLRP3 inflammatory bodies, thus slowing down the evolution of non-alcoholic steatohepatitis [88]. By weakening the inflammatory response through the interaction with the TGR5 receptor, BAs contribute to the reduction in the advancement of MAFLD [89].

The addition of Fucoidan (FUC) and galactooligosaccharides (GOS) two bioactive compounds derived from seaweed (algae polysaccharides), showed promising results in improving dyslipidemia, BSH activity, and composition of BAs by increasing the abundance of gut microbiota *Enterobacter* that is involved in BSH-related activities. Furthermore, FUC and GOS increased the expression of CYP7A1 in the liver, leading to decreased serum cholesterol levels and alleviation of hepatic steatosis [90]. Another noteworthy plant polysaccharide, *Gracilaria lemaneiformis* polysaccharide (GLP, plant polysaccharides) could alleviate hyperlipidemia and reduce liver fat accumulation by modulating the abundance of specific gut bacteria such as *Bacteroides*, *Ruminococcus_1* and *Lactobacillus* to increase the conversion of primary BAs to secondary BAs. Simultaneously, GLP promoted the reduction of hydrophobic BAs CDCA and DCA through the upregulation of *Prevotellaceae_UCG-001*, *Corprococcus_1*, and *Alistipes* while increasing the levels of hydrophilic BAs such as UDCA and TUDCA via the stimulation of *Roseburia* and *Lachnospiraceae_NK4A136_group*. Mechanically, GLP could enhance the expression of CYP7A1 by activating liver X receptor alpha (LXRα) to accelerate the cholesterol conversion and also could reduce cholesterol synthesis through AMPK-Sterol regulatory element-binding protein-2 (SREBP-2)-HMGR-related signaling pathway [65]. PL-S2, polysaccharides derived from Radix Puerariae lobatae (plant polysaccharides), reversed HFD-induced microbiota changes and modulated BAs metabolism further ameliorating lipid metabolism disorder and hepatic tissue injury. The mechanism behind this effect involved the upregulation of proteins in the FXR signaling pathway, including CYP7A1, bile salt export pump (BSEP), and multidrug resistance-associated protein 2 (MRP2). These proteins were crucial for the conversion of cholesterol into BAs by CYP7A1 and subsequent transport into bile through BSEP and MRP2, at the same time increasing the activity of FXR by enhancing PPAR and LXR which were related to anti-inflammation effects [91]. Ganoderma lucidum polysaccharide peptide (GLPP, fungi polysaccharides) upregulated key enzymes including CYP7A1 and CYP8B1, as well as activated FXR and SHP, while downregulating the liver fibroblast growth factor receptor 4 (FGFR4)/β-Klotho receptor complex to reduce its inhibition on CYP7A1 in ob/ob spontaneous hepatic steatosis mice and ApoC3 transgenic hyper-triglyceridemic mice, increasing the de novo synthesis rate of BAs, which proved that GLPP treated MAFLD via regulating BAs metabolism dependent on FXR-SHP/FGF pathway [92]. Inulin as polysaccharides (plant polysaccharides) released liver lipid deposition in HFD-induced MAFLD mice. Inulin decreased FXR antagonist secondary BAs and dramatically increased the strongest agonist of FXR CDCA. This shift in BA composition reversed the decline in intestinal FXR-FGF15 signaling and downregulated the expression of key transporters involved in BA absorption, including apical sodium-dependent bile acids transporter (ASBT) in the gut, Na^+^-taurocholate co-transporting polypeptide (NTCP) and organic anion transport protein (OATP) in liver to decrease total absorption of BAs, enhancing the excretion of BAs from the body [93]. These findings underscored the potential of polysaccharides as therapeutic interventions to modulate BA metabolism by targeting key regulatory pathways and transporters and improving the pathogenesis of MAFLD.

## 4. Conclusions and Prospection

MAFLD, a metabolic disorder, has witnessed a rapid increase in prevalence in recent years, primarily associated with obesity, T2DM, and metabolic disturbances. The precise underlying mechanism for the heightened risk of MAFLD remains unclear, and the heterogeneity within the affected population has hindered the establishment of universally recognized therapeutic interventions [94]. However, polysaccharides, which exist abundantly in everyday food, have demonstrated a range of beneficial biological activities, particularly in promoting gut microbiota balance and enhancing intestinal barrier integrity. Consequently, polysaccharides hold significant potential as a crucial component of novel dietary therapies for managing MAFLD.

This narrative review explores the impact of polysaccharides from diverse sources on MAFLD, exerting their effects through direct modulation of various gut microbiota populations or their metabolites. Due to their indigestible nature, polysaccharides are broken down by intestinal bacteria that secrete CAZymes. Through regulation of the gut-liver axis, polysaccharides have the ability to modulate the composition and diversity of gut microbiota, effectively reversing the intestinal dysbiosis induced by HFD. The resultant metabolites derived from gut microbiota, such as LPS, SCFAs, and BAs, collectively referred to as metabolic regulators, likely serve a crucial purpose in linking the therapeutic potential of polysaccharides to the progression of MAFLD. Specifically, polysaccharides maintain the balance of microbiota, intestinal barriers, and immune function, thereby reducing the translocation of bacterial product LPS into the bloodstream and preventing liver damage. Within the intestinal tract, polysaccharides are decomposed into SCFAs, promoting the proliferation of SCFAs-producing bacteria and enhancing SCFAs-related signaling pathways. Besides, the increasing levels of secondary BAs and hydrophilic BAs suggest the involvement of BAs-related, particularly FXR signaling pathways, in the mitigation and therapy of MAFLD.

In summary, the modulation of gut microbiota through the gut-liver axis appears to be a key mechanism by which polysaccharides combat MAFLD. However, the current comprehension of the specific mechanisms underlying the beneficial impacts of polysaccharides in improving MAFLD remains relatively restricted. Most studies have been conducted solely in rodent models, with a dearth of clinical and practical application research. Polysaccharides, characterized by their distinct structures, exhibit unique probiotic effects that are multi-targeted and synergistic in the treatment of MAFLD. Therefore, it is necessary to accurately investigate the precise relationship between the polysaccharide structure and specific gut microbiota to harness their full probiotic potential in preventing and treating MAFLD. An in-depth exploration of microbiomics and metabolomics changes induced by polysaccharides, verified in cellular and organoid models, will provide a solid foundation for future clinical applications. Furthermore, this research may guide the exploitation of novel polysaccharide-based products with therapeutic significance, offering a promising approach for the intervention and investigation of metabolic diseases.

## Figures and Tables

**Table 1:** Summary of probiotic effect of polysaccharides in metabolic associated fatty liver disease (This comprehensive table provides a detailed overview of various polysaccharides, including their sources and composition, along with different types of the MAFLD model in intervention. Each entry is accompanied by relevant references for further exploration. Furthermore, this table delves into the regulation of microbiota in the disease model and highlights the corresponding changes in relevant indexes. Additionally, it sheds light on the intriguing exploration of the possible mechanisms through which these polysaccharides exert their therapeutic effects in ameliorating MAFLD.).

Polysaccharides	Source	Composition	Disease Model	Alteration of Microbiota	Results or Mechanisms	References
HSM (20 mg/kg)	*Hirsutella sinensis*	β-glucans, heteroglycans, cordyglucans,	HFD 12 weeks male mice	*P. goldsteinii*, *Flintibacter butyricus*, *Intestinimonas*↑ *Pseudomonas aeruginosa, Escherichia coli*, *Shewanella algae*↓	(1) BW, LW, visceral fat mass, histopathological hepatic steatosis↓ (2) lipogenesis, lipid transport and uptake, TG↓, hepatic β-oxidation↑ (3) FBG, INS, HOMA-IR↓, insulin sensitivity↑ (4) thermogenesis, UCP-1↑ (5) LPS-inflammation↓, IL-10, ZO-1↑	[51]
GFP (150/400 mg/kg)	*Grifola frondosa*	D-mannose, D-glucose, D-galactose	HFD 8 weeks rats	Helicobater, *Intestinimonas*, *Barnesiella*, *Parasutterella*, *Ruminococcus*, *Flavonifracter*↑ *Firmicutes*/*Bacteroidetes* (F/B) ratio, Clostridium-XVIII, *Butyricicoccus*, *Turicibacter*↓	(1) BW, histopathological hepatic steatosis↓ (2) TG, TC, FFA, AST, ALT↓, HDL-c, fecal BAs↑ (3) MDA↓, GSH-Px, T-SOD↑ (4) ACAT2, GS, CYP4A1, ACC, TNF-α, SOCS2↓, AMPKα, PPARγ, CYP7A1, Acox1, SOD, CAT, BSEP↑	[52,53]
WGHP (600 mg/kg)	*Walnut green husk*	galacturonic acid (52.12%), arabinose (15.96%), galactose (14.44%), glucose (8.31%), rhamnose (6.41%), xylose (1.48%), Mannose (0.66%), glucuronic acid (0.6%), fucose (0.2%)	HFD 50 days rats	*Prevotellaceae*, *Allobaculum*↑ F/B ratio, *Lactobacillaceae*, *Lachnospiraceae*↓	(1) BW, LW, visceral fat mass, histopathological colonic damage, hepatic steatosis↓ (2) TC, TG, LDL-C, NEFA, AST, ALT↓, HDL↑ (3) UCP-1, NRF1, NRF2, Cidea, PRDM16, CPT1, PRDM16, TMEM26, PCG-1α, CD137↑ (3) MDA, PPARα, P-JNK/JNK, TNF-α, MCP-1↓, GSH-Px, T-SOD, Nrf2↑, ZO-1, Occludin, MUC2↑ (4) acetic acid, propionic acid, butyric acid, valeric acid↑	[54,55]
LBP (50 mg/kg)	*Lycium barbarum*	mannose:rhamnose:glucose:galactose:arabinose = 1.00:0.93:12.55:0.31:0.53	HFD 8 weeks Rats HFD 12 weeks male mice	*Deferribacteracean*, *Lactobacillus*↑ *Enterococcaceae*, *Proteobacteria*, F/B ratio↓	(1) BW, LW, histopathological colonic and ileac damage, hepatic steatosis↓ (2) TC, TG, leptin, LDL, FFA↓HDL↑ (3) FBG, INS, HOMA-IR↓, glucose tolerance, insulin sensitivity↑ (4) LPS-TLR4- NF-κB-inflammation↓, ZO-1, Occludin↑ (5) acetic acid, butyric acid, valeric acid↑	[56,57]
Lentinan (500 mg/kg)	*Shiitake mushrooms*	β (1,3)/β (1,6)-glucan	HFD 15 weeks male mice	*Actinobacteria*, *Firmicutes↑* *Proteobacteria*, *Epsilonbacteraeota*↓	(1) histopathological hepatic steatosis↓ (2) iNOS↓, HO-1, NQO1, Gclc↑ (3) LPS-TLR4- NF-κB-inflammation↓, IL-10, Arg1, ZO-1, Occludin↑ (4) NFκB-PTP1B-Akt-GSK3β↑	[58]
*Salvia miltiorrhiza* polysaccharide (50 mg/kg)	*Salvia miltiorrhiza bunge*	-	HFD 6 weeks male mice	*Bacteroides*, *Lactobacillus*, *Parabacteroides↑* *Cyanobacteria*, F/B ratio↓	(1) BW, LW, histopathological hepatic steatosis↓ (2) TC, TG, LDL-C, FFA, AST, ALT↓, HDL↑ (3) FBG, INS, HOMA-IR↓, insulin sensitivity↑ (4) LPS-TLR4- NF-κB-inflammation↓ (5) ACC, FAS, SREBP-1c↓, PPARα, Cpt1α↑ (6) acetate, butyrate↑	[59]
SMRR (10/20 mg/kg)	*Salvia miltiorrhiza bunge*	galacturonic acid:arabinose:galactose:rhamnose:glucose = 17.9:1.3:1.7:1.2:1	HFD 8 weeks male mice	*Ruminococcus gnavus*, Clostridium_cocleatum, Bifidobacterium_pseudolongumBifidobacterium, Lactobacillus, Leuconostoc↓	(1) BW, histopathological jejunal and colonic damage, hepatic steatosis↓ (2) TC, TG, LDL-C, HDL -C, NEFA, AST, ALT↓ (3) LPS, IL-6, IL23↓IL2, IL10, TGF-β↑	[60]
MDG-1 (2‰, 4‰, 8‰)	*Ophiopogon japonicus*	inulin-type β-D-fructan, trace of α-D-GLc	HFD 8 weeks male mice	*Alistipes*, *Ruminiclostridium*, *Ricenella*, *Butyricimonas*, *Roseburia*↑ F/B ratio, *Lactococcus*, *Enterorhabdus*, *Turicibacter*, Clostridium-sensu-stricto-1, *Tyzzerella*, *Oscillibacter*↓	(1) BW, histopathological hepatic steatosis↓ (2) TC, TG, AST, ALT↓ (4) IL-1β, IL-4, TNF-α, CD68↓ IL-10↑ (5) SREBP-1c, FAS, ACC-1, PPARγ↓, acetic acid, valeric acid, GPR41/43-AMPK↑	[61]
APS (4% in finial concentration)	*Astragalus mongholicus Bunge*	rhamnose (1.6%), arabinose (23.39%), xylose (0.84%), glucose (70.55%), galactose (3.61%)	HFD 8 weeks male mice	*Desulfovibrio vulgaris* (acetate producing bacteria)↑ F/B ratio↓	(1) BW, histopathological hepatic steatosis↓ (2) TG, ALT, serum insulin↓ (3) IL-1β, IL-6↓ (4) GCK, FASN, CD36↓, PPARα, Cpt1α↑ (5) acetate↑	[62]
mAPS (200 mg/kg)	*Astragalus mongholicus Bunge*	glucose (84.86%), arabinose (4.49%), galactose (3.92%), ribose (3.26%)	HFD 6 weeks rats	*Proteobacteria*, *Episilonbacteria* ↑ F/B ratio↓	(1) BW, histopathological hepatic steatosis↓ (2) TC, TG, LDL-C, AST, ALT↓, HDL↑ (3) HOMA-IR↓ (4) SREBP-1↓, AMPK, PPAR-α↑ (5) LPS-TLR4-NF-κB-NLRP3, inflammation↓, ZO-1, Occludin↑	[63]
FUC + GOS (100 mg + 800 mg/kg)	*seaweed*	FUC: sulfate (27.8%), fucose (20.3%), GOS: trisaccharides (37.7%), tetrasaccharides (21.7%), disaccharides (18.58%), pentose (12.1%)	HFD 8 weeks rats	*Proteobacteria*, *Verrucomicrobia Enterobacter*↑ *Actinobacteria*, *Cyanobacteria*, F/B ratio↓	(1) BW, histopathological hepatic steatosis↓ (2) TC, LDL-C, TBA↓ (3) CYP7A1, BSH↑, LPS↓	[64]
GLP (60, 225 mg/kg)	*Gracilaria lemaneiformis* (0.1 mL/10 g)	Galactose:glucose:fucose:mannose = 9.16:6.57:1.00:0.61	High-fat and high- cholesterol diet 40 days male mice	*Bacteroides*, *Ruminococcus_1*, *Lactobacillus*, *Prevotellaceae_UCG-001*, *Corprococcus_1*, *Alistipes*, *Roseburia*, *Lachnospiraceae_NK4A136_group*↑	(1) BW, LW, histopathological hepatic steatosis↓ (2) TC, TG, FFA↓ (3) Primary BAs, hydrophobic BAs↓, Secondary BAs, hydrophilic BAs↑ (4) SREBP-2-HMGR↓, AMPKα, LXRα- CYP7A1↑	[65]

In the table, ‘↓’ means down-regulation after administration of polysaccharides; ‘↑’ means up-regulation after administration of polysaccharides. Abbreviations: HSM, *Hirsutella sinensis* mycelium polysaccharides; GFP, Grifola frondose polysaccharides; WGHP, Walnut green husk polysaccharides; LBP, *Lycium barbarum* polysaccharides; SMRR, *Salviae miltiorrhizae* Radix et Rhizoma; APS, Astragalus polysaccharides; mAPS, Astragalus mongholicus polysaccharides; FUC, Fucoidan; GOS, galactooligosaccharides; GLP, *Gracilaria lemaneiformis* polysaccharides.
